# ASSOCIATION BETWEEN OLDER PARENTS’ INFORMATION SHARING AND THEIR INTERGENERATIONAL AMBIVALENCE

**DOI:** 10.1093/geroni/igac059.2336

**Published:** 2022-12-20

**Authors:** Noriko Toyokawa, Nancy Darling, Teru Toyokawa

**Affiliations:** Southern Oregon University, Ashland, Oregon, United States; Oberlin College, Oberlin, Ohio, United States; California State University San Marcos, San Marcos, California, United States

## Abstract

The current study examined (1) the relation between older parents’ information sharing of their life issues with their children as the primary helpers (helper children) and parents’ intergenerational ambivalence and (2) the effect of parents’ reasons for not sharing the information with the helper children on parents’ intergenerational ambivalence. Older parents (N=268, Mage =76, SD=5.41, range 65-94, female=59%) participated in an online survey and responded to how much they openly shared information on 10 issues of their lives developed based on a focus group study (Toyokawa et al., 2021). A series of one-way ANOVAs, followed by Bonferroni posthoc analysis, revealed that compared to parents who openly shared information on life issues and did not perceive the issues were not applicable for their lives, parents who did not openly share information reported significantly lower levels of intergenerational ambivalence on the issues of pain management, F(2, 265)=7.51, p=.0007, social partners, F(2,265)=9.50. p=.0001,what they do with other children, F(2,256)=7.49, p=.0007, financial management, F(2,265)=3.80, p=.0234, and their plan for the end-of-life, F(2,256), p=.004. A series of t-tests showed that parents who did not share the information with the helper children because of avoiding unwanted interventions reported significantly higher scores of intergenerational ambivalence than parents who did not share the information to prevent the helper children from being worried about them in eight issues. The findings suggest parents maintain their autonomy by limiting the helper children’s monitoring ability. Possible reasons for older parents’ managing issue-specific information sharing will be discussed.

